# ^4^He Thermophysical Properties: New Ab Initio Calculations

**DOI:** 10.6028/jres.112.006

**Published:** 2007-04-01

**Authors:** John J. Hurly, James B. Mehl

**Affiliations:** National Institute of Standards and Technology, Gaithersburg, MD 20899-8360; K T Consulting, Inc., P.O. Box 307, Orcas, WA 98280

**Keywords:** helium, second virial, theoretical interatomic potential, thermal conductivity, thermophysical standards, transport properties, viscosity

## Abstract

Since 2000, atomic physicists have reduced the uncertainty of the helium-helium “ab initio” potential; for example, from approximately 0.6 % to 0.1 % at 4 bohr, and from 0.8 % to 0.1 % at 5.6 bohr. These results led us to: (1) construct a new inter-atomic potential *ϕ*_07_, (2) recalculate values of the second virial coefficient, the viscosity, and the thermal conductivity of ^4^He from 1 K to 10,000 K, and (3), analyze the uncertainties of the thermophysical properties that propagate from the uncertainty of *ϕ*_07_ and from the Born-Oppenheimer approximation of the electron-nucleon quantum mechanical system. We correct minor errors in a previous publication [J. J. Hurly and M. R. Moldover, J. Res. Nat. Inst. Standards Technol. 105, 667 (2000)] and compare our results with selected data published after 2000. The ab initio results tabulated here can serve as standards for the measurement of thermophysical properties.

## 1. Introduction

In 2000, Hurly and Moldover published a comprehensive report on the application of fundamental physics to the calculation of the thermophysical properties of low-density helium [1]. The present paper is an extension to and update of parts of that paper. We developed a new model potential for the interaction of helium atoms, *ϕ*_07_(*r*), based on the most recent theoretical values of *ϕ*(*r*). This potential was used to calculate several important properties of ^4^He: The density virial coefficient *B*(*T*) and its first two temperature derivatives, the zero-density viscosity, and the zero-density thermal conductivity.

Our improved potential and calculations have significantly reduced the uncertainty of the thermophysical properties of helium. For example, at 300 K, the uncertainty of the second virial coefficient is now 1/7 of that reported in Ref. [1] and the uncertainty of the thermal conductivity is 1/3 of that reported in Ref. [1].

The new potential includes the diagonal correction to the Born-Oppenheimer model (DBOC). In addition to the use of this correction, recent discussions of the adiabatic model [2,3] recommend the use of atomic, rather than nuclear, masses in the calculations of atomic interactions. We have examined the sensitivity of the thermophysical properties to this replacement, as well as to the DBOC and to the uncertainties of the theoretical calculations of *ϕ*.

In the temperature range 3 K to 933 K, helium gas thermometry [4] played a leading role in the formation of the internationally accepted temperature scale, ITS-90. Subsequently, improved gas thermometry has measured *T* − *T*_90_, the differences between the thermodynamic temperature and ITS-90. Thus improved gas thermometry [5] may lead to a future, improved tem perature scale. Each form of gas thermometry (constant volume, dielectric, acoustic) requires the extrapolation of measured gas properties to zero pressure, where gases become “ideal.” In this work, we use fundamental principles to calculate the second density virial coefficient of helium *B*(*T*) and the second acoustic virial coefficient of helium *β_a_*(*T*) with smaller uncertainties than can be achieved by direct measurements. Our tabulated values for *B*(*T*) and *β_a_*(*T*) can be used to constrain the extrapolations to zero pressure; thereby leading to more accurate values of the thermodynamic temperature. Acoustic gas thermometry also requires accurate values of the thermal conductivity, which we have tabulated for helium. Recently, May *et al.* [6] have shown how to combine ab initio values of the viscosity of helium with comparatively simple viscosity-ratio measurements to obtain values of the thermal conductivity of argon that are more accurate than can be achieved by direct measurements. Thus, our tabulation of the viscosity of helium will also facilitate more accurate argon-based acoustic thermometry. Finally, we mention programs to redetermine the Boltzmann constant [7] and to develop an atomic standard of pressure [8] based on accurate measurements of the dielectric constant of helium at the temperature of the triple point of water (*T*_TPW_ = 273.16 K). Both of these programs will benefit from the reduced uncertainties of *B*(*T*).

In the following sections, we first describe the potential and the way it was developed. We summarize the quantum-statistical formulas used for calculating the thermophysical properties of interest, then describe the numerical procedures used for the calculations. We conclude with some comparisons of our theoretical thermophysical properties with recent experimental results.

Standard notation conventions are followed in this paper. All quantum-mechanical formalism is expressed in atomic units except when noted otherwise. Interaction potentials are expressed in hartrees in the formalism, but converted to temperature units (K) for comparison with relevant literature. The CODATA-2002 values of the fundamental constants [9] were used in all calculations.

## 2. Model Potential *ϕ*_07_

The potential model is expressed as the sum of a repulsive term and an attractive term

$$\phi_{07}(r)=\{\begin{array}{ll} \phi_{\text{rep}}(r)+\phi_{\text{att}}(r), & r_{0}\leq r<\infty \\ \phi_{\text{rep}}(r_{0})+\phi_{\text{att}}(r_{0}), & 0\leq r<r_{0}, \end{array}$$ (1)

$$\phi_{\text{rep}}(r)=A\exp\left(\sum_{n=-2}^{2}a_{n}r^{n}\right),$$ (2)

$$\phi_{\text{att}}(r)=-A\sum_{n=3}^{8}\frac{f_{2n}(r)C_{2n}}{r^{2n}}\left[1-\left(\sum_{k=0}^{2n}\frac{{\left(\delta r\right)}^{k}}{k!}\right)e^{-\delta r}\right].$$ (3)

In these equations the cut-off radius *r*_0_ = 0.3 bohr is chosen to exclude the unphysical behavior of the potential model at small *r*; *A* = 1 hartree (*E_h_*) defines the units; the *a_n_* and *δ* are fit parameters; the *C*_2_*_n_* are fixed parameters; and the functions *f*_2_*_n_* account for relativistic retardation. The attractive part of the potential is the sum of multipole attractive terms multiplied by the universal damping functions of Tang and Toennies [10].

The dipole-dipole and higher multipole parameters *C_n_* for *n* ≤ 10 (Table 1) are fixed at the values calculated by Zhang *et al.* [11]. The coefficients *C_n_* for helium with the mass of ^4^He were used in all calculations except those which investigated corrections to the Born-Oppenheimer model. The coefficients *C*_2_*_n_* for *n* > 5 were estimated using the three-term recursion formula of Thakkar [12].

Zhang *et al.* [11] include an extensive tabulation of previous calculations for comparison. The fractional differences between the fixed-nucleon parameters of Zhang *et al.* and those of Bishop and Pipin [13] are 2.6 × 10^−8^ for *C*_6_, 1.7 × 10^−7^ for *C*_8_, and −9.5 × 10^−7^ for *C*_10_. If these fractional differences are taken as estimates of uncertainties, the total uncertainty in the potential is less than 3 × 10^−8^ K, and the total fractional uncertainty is less than 5 × 10^−8^, for *r* > 10 bohr.

A further, and more significant, source of uncertainty is the extrapolation formula used to estimate *C*_2_*_n_* for *n* > 5 from the lower-*n* values of *C*_2_*_n_*. Thakkar [12] recommends the use of either his Eqs. (29) or (33), with the latter more appropriate for helium (based on the value of $C_{6}C_{10}C_{8}^{2}$). With the alternative formula, the estimated values of *C*_12_, *C*_14_, and *C*_16_ are 1.3 %, 5.2 %, and 13 % larger. If these differences are used as estimates of the uncertainties of the corresponding potential contributions, the total uncertainty in the potential is less than 4 × 10^−5^ K, and the total fractional uncertainty is less than 6 × 10^−5^, for *r* > 10 bohr.

In principle, the use of the Tang-Toennies damping terms [10] is an additional source of uncertainty. However, these functions differ from unity only for *r* ~ 10 bohr and below, where the quality of the fit potential can be judged directly by comparison with theoretical potentials. (See Fig. 2.)

The retardation functions *f*_6_, *f*_8_, and *f*_10_ have been calculated by Chen and Chung [14]. Their results for *f*_6_ are in excellent agreement with the calculations of Jamieson *et al.* [15], whose results differ from those of Chen and Chung by a maximum fraction 1.5 × 10^−5^. The retardation functions satisfy *f*_2_*_n_*(0) = 1; *f*_6_ decreases to ½ for *r* ≈ 500 and approaches 328.47/*r* for large *r*; *f*_8_ decreases to ½ for *r* ≈ 660 and approaches 420.62/*r* for large *r*; and *f*_10_ decreases to ½ for *r* ≈ 810 and approaches 508.43/*r* for large *r*. The functions *f*_2_*_n_* have the effect, for example, of converting the dipole-dipole interaction from a 1/*r*^6^ dependence to a 1/*r*^7^ dependence. Retardation has, at most, a marginal effect on all terms except the dipole-dipole term. At *r* = 660 bohr, the ratio *C*_8_*f*_8_/*r*^8^ to *ϕ*_07_ is less than 3 × 10^−5^; similarly, at *r* = 810 bohr, the ratio of *C*_10_ *f*_10_/*r*^10^ to *ϕ*_07_ is less than 4 × 10^−10^. Accordingly, the factors *f*_12_, *f*_14_, and *f*_16_, were safely approximated as unity. The code for computing the potential uses cubic spline interpolation of the results of Chen and Chung [14] for *f*_6_, *f*_8_, and *f*_10_.

The parameters *δ* and *a_j_*, −2 ≤ *j* ≤ 2 were determined by fitting the potential model (1)–(3) to selected theoretical values weighted to account for their estimated uncertainties. The retardation functions *f*_2_*_n_* were set to unity in these fits. Several fits were made. The first was to the selected data set described below, and will be referred to as *ϕ*_07_. The second and third fits accounted for the uncertainties of the theoretical values, also described below. The corresponding potentials are designated *ϕ*_07±_. An additional fit was made to the potential values without applying the diagonal Born-Oppenheimer correction [16]. The corresponding potential is *ϕ*_nboc_. The values of *δ* and the *a_j_* determined by these fits are listed in Table 2.

### 2.1 Theoretical Values of *ϕ*

Table 3 lists values of the potential *ϕ* and their uncertainties based on our review of the recent literature [17–26]. The values selected for determination of *ϕ*_07_(*r*) represent a compromise based on availability of calculations at each *r*, the uncertainty claimed by the authors, and the internal agreement of various calculations for nearby *r*. The theoretical values were obtained within the Born-Oppenheimer model for fixed nuclear separations. Uncertainties were assigned to each of the selected values. When only a single datum was available, the authors’ uncertainty estimate was used, provided that it was consistent with neighboring values; otherwise the uncertainty was adjusted upward. When several values were available at an *r*-value, generally the unweighted mean and standard deviation of the more recent calculations was used. The upper-bound potentials of Komasa [19] were used only at small *r*, where they are in excellent agreement with the quantum-Monte-Carlo calculations of Ceperley and Partridge [17], which have much larger uncertainties.

The diagonal Born-Oppenheimer correction calculations of Komasa, Cencek and Rychlewski [16] were interpolated using a cubic spline and added to the fixed-nucleon potentials.

Relativistic [27] (+15.4 mK) and radiative [28] (−1.3 mK) corrections to the potential have recently been evaluated only at *r* = 5.6 bohr. Without additional results at other *r* we decided, for consistency, to omit these corrections from the determination of *ϕ*_07_. The sum of these corrections is small compared with the scatter of the *r* = 5.6 bohr potentials in Fig. 3, but of the same order as the assigned uncertainty.

The model potential defined by Eqs. (1)–(3) was fit to the sum of two quantities, the selected potentials and the corresponding DBOC. The input potentials were weighted by the inverse squares of the uncertainties *U*(*ϕ*) in the fit. The coefficients determined in the fit are listed in Table 2. The variance of the fit residuals in the determination of *ϕ*_07_ was 0.6.

**Fig. 1:**
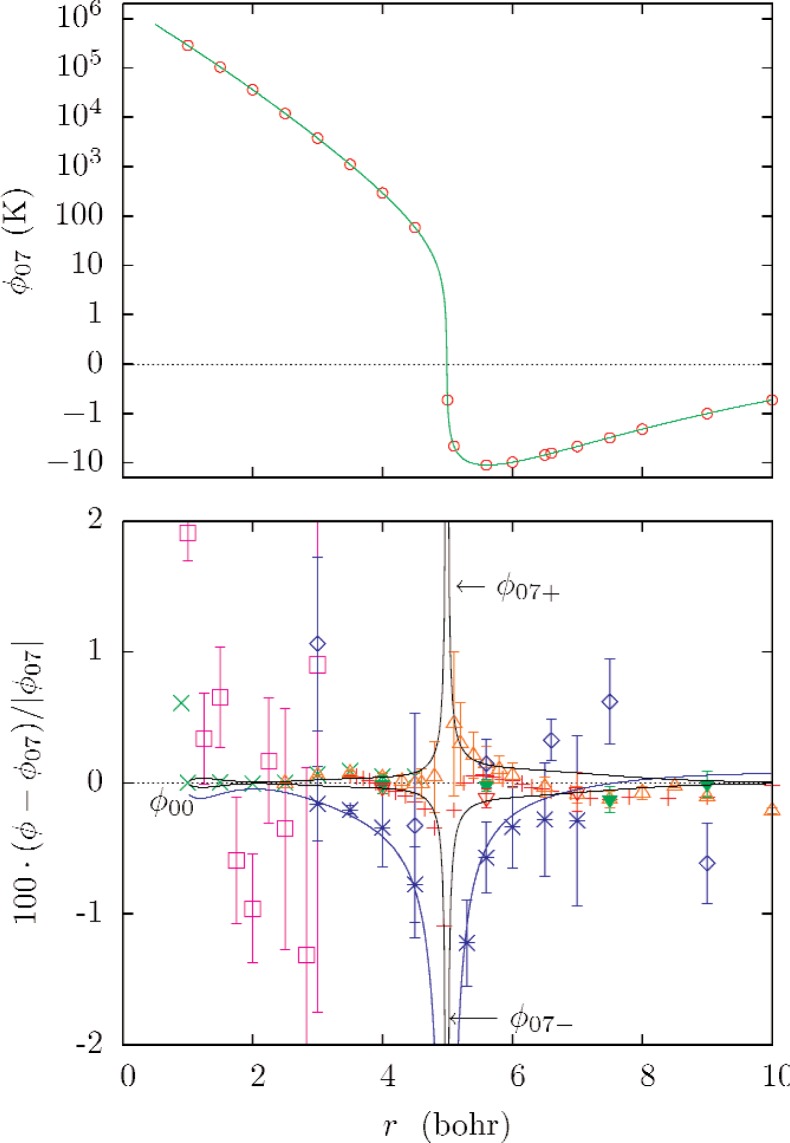
Top: The model potential *ϕ*_07_ (solid line) and theoretical values of *ϕ* (open circles) used in its determination. (The vertical scale is proportional to sinh^−1^(5*ϕ*/K), which is approximately logarithmic for large *ϕ* and linear for small |*ϕ*|.) Bottom: Fractional differences between theoretical values of *ϕ* and the model potential *ϕ*_07_, with error bars as assigned by the authors (when available). The data sources are □ [17], * [18], × [19], + [20], Δ [23], ⋄ [24], ▼ [25], ∇ [26]. The potentials *ϕ*_07±_ and *ϕ*_00_ [1] are shown as solid lines.

**Fig. 2:**
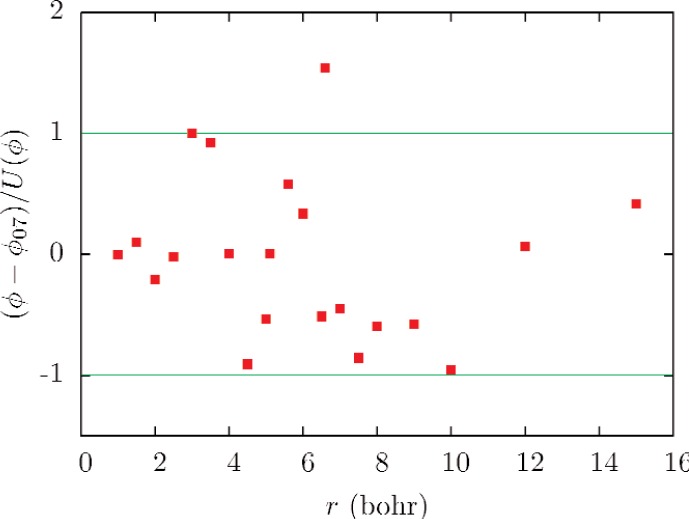
Differences between the theoretical potential values used in fitting *ϕ*_07_ and the potential, divided by the uncertainties of the potential values (See Table 3).

**Fig. 3:**
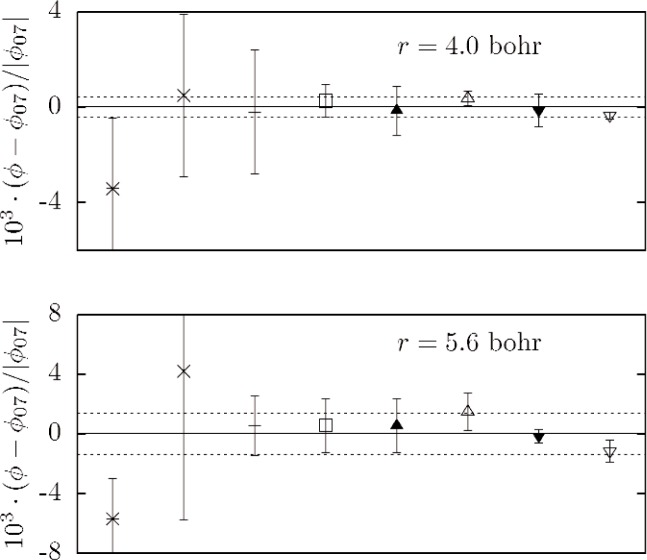
Comparisons of theoretical values of *ϕ* with the (unretarded) model potential *ϕ*_07_. The plotted values, arranged chronologically by date of publication, are from the following sources: * [18], × [19] (upper bound), + [20], □ [21], ▲ [22], Δ [23], ▼ [25], ∇ [26]. The dotted lines represent *ϕ*_07±_. These bounds encompass the eight values of *ϕ*(*r*) published since 1999 [19–23,25,26], or overlap the authors’ *k_u_* = 1 uncertainty estimates. The values of *ϕ*_07_ less the diagonal Born-Oppenheimer correction are (292.64 ± 0.13) K at 4 bohr and (−10.996 ± 0.015) K at 5.6 bohr.

The upper part of Fig. 1 shows the potential *ϕ*_07_ and the selected data used in its determination. The lower part of Fig. 1 and Fig. 3 show fractional differences between many recent theoretical potentials and *ϕ*_07_. Figure 2 shows the normalized residuals (*ϕ* − *ϕ*_07_)/*U*(*ϕ*).

To assess the uncertainty of *ϕ*_07_ and the propagation of this uncertainty into computed thermophysical properties, the potentials *ϕ*_07+_ and *ϕ*_07−_ were developed. The potential was refitted to theoretical potentials shifted by their uncertainties, that is, to *ϕ* + Δ*ϕ*_DBOC_ ± *U*(*ϕ*). Similarly, the effects of the diagonal Born-Oppenheimer correction were assessed by determining the potential *ϕ*_nboc_ through fits to the theoretical *ϕ* values without adding the correction.

The uncertainty of *ϕ*_07_ is difficult to quantify. Figure 2 shows that all but one of the theoretical potentials used in fitting *ϕ*_07_ differs from *ϕ*_07_ by less than the corresponding uncertainty, consistent with the fit variance of 0.6. Figure 3 shows that all of the theoretical values at *r* = 4 bohr and *r* = 5.6 bohr that were used in the fit either fall in the range between *ϕ*_07−_ and *ϕ*_07+_ or have uncertainties overlapping this range. These observations suggest that the uncertainty in *ϕ*_07_ should be interpreted as having a large coverage factor [29] *k_u_* ≈ 2. Table 4 summarizes the properties of the potentials used in this work, and the bound state energies (for angular momentum index *ℓ* = 0) determined from the potential.

## 3. Atomic Interactions

The thermophysical properties of helium can be evaluated using the formalism of quantum statistical mechanics. In particular, the virial coefficient of the equation of state, the viscosity, and the thermal conductivity can be expressed in terms of the phase shifts associated with the interaction of a pair of helium atoms. The theory and equations used in determining the thermophysical properties are summarized in Sec. 3.1. The following section 3.2 describes the computational techniques used to determine the thermophysical properties.

### 3.1 Formalism

The interaction of two atoms with a spherically symmetric potential *ϕ*(*r*) is described by a quantum mechanical wave function Ψ*_ℓ_* (*r*)*Y_ℓm_*/*r*, where *r* is the separation distance and *Y_ℓm_* is a spherical harmonic. The radial function satisfies

$$\left\{\frac{d^{2}}{dr^{2}}-\frac{\ell (\ell +1)}{r^{2}}-\frac{2\mu }{m_{e}}[\phi (r)-E]\right\}\Psi_{\ell }(r)=0,$$ (4)

where *µ* is the reduced mass of the He-He system, *m_e_* is the electron mass, lengths are expressed in units of the Bohr radius *a*_0_, and energies are expressed in units of hartree (*E_h_*).

The solutions to Eq. (4) fall into three ranges. For small *r*, where the potential is much larger than the angular-momentum term, the solutions must be determined numerically. In the second region of intermediate *r*, the potential is negligible but the angular momentum term is significant, so Eq. (4) takes the form

$$\left[\frac{d^{2}}{dr^{2}}-\frac{\ell (\ell +1)}{r^{2}}+\kappa^{2}\right]\chi_{\ell }(r)=0,$$ (5)

where

$$\kappa^{2}=(2\mu /m_{e})E,$$ (6)

that is, *κ* is just the wave number *k* = *κ*/*a*_0_ in atomic units. The general solution of Eq. (5) is

$$\chi_{\ell }=\kappa rA_{\ell }[\cos\delta_{\ell }\cdot j_{\ell }(\kappa r)-\sin\delta_{\ell }\cdot y_{\ell }(\kappa r)],$$ (7)

where *j_ℓ_*(*ξ*) and *y_ℓ_*(*ξ*) are spherical Bessel and Neumann functions. For large *κr* the asymptotic form of *χ_ℓ_*. is

$$\chi_{l\underset{r\to \infty }{\to }}A_{\ell }\sin(\kappa r-\ell \pi /2+\delta_{\ell }),$$ (8)

which can be recognized as the solution to Eq. (4) in the third region, where both the potential and the angular momentum term are negligible. The thermophysical properties of interest depend on the phase shifts *δ_ℓ_*(*E*). The virial coefficient of ^4^He depends on the sum

$$S(\kappa )=\sum_{\ell =0,2,4,\ldots }^{\infty }\left(2\ell +1\right)\delta_{\ell }(\kappa ).$$ (9)

The convergence of this sum is discussed in the next section. The viscosity and thermal conductivity depend on the quantum cross-sections [30] which are expressed in terms of much more rapidly converging sums.

### 3.2 Numerical Techniques

Numerical solutions of the radial equation (4) were determined with Numerov’s method using an integration step size

$$h_{0}=2\times 10^{-5}\cdot E^{-1/3}.$$ (10)

This step size was determined empirically to insure that phase shifts obtained with step sizes *h*_0_/2 or *h*_0_/4 did not differ from those determined with step size *h*_0_ within the error criterion |Δ*δ_ℓ_*| < 10^−9^. Calculations were made for a series of discrete energies in the range 10^−11^ ≤ *E*/*E_h_* ≤ 1. The discrete energies were distributed uniformly on a logarithmic scale.

For each discrete energy, Eq. (4) was integrated upward in *r*, for *ℓ* = 0, 2, … *ℓ*_1_. A series of nodes of Ψ*_ℓ_* (*r*) were found at coordinates *r_n_*, *n* = 1, 2, …. The phase shifts at node *n*, *δ_ℓ,n_*, defined by

$$\tan\delta_{\ell ,n}=j_{\ell }(\kappa r_{n})/y_{\ell }(\kappa r_{n}).$$ (11)

were determined successively. The asymptotic phase shift as *r_n_*→∞ was obtained when the phase shifts evaluated at a series of nodes agree to within the preset convergence criterion. Convergence was accelerated by using the semi-classical (JWKB) approximation [31,32]. The convergence criterion was that the standard deviation of three successive values of δ*_ℓ,n_* was less than 10–9. The maximum angular momentum index *ℓ*_1_ was the minimum of either 1000 or the index when |δ*_ℓ_*| became less than 10–9.

Equation (11) only determines the phase shift within an additive multiple of π. Two conditions were used to get the total phase shifts needed in the sum (9). (1) The limiting values were lim*_E_*_→0_*δ*_0_(*E*) = π and lim*_E_*_→0_*δ_ℓ_* (*E*) = 0 for *ℓ* > 0; and (2) *δ_ℓ_*(*E*) is a continuous function of *E* [33].

**Fig. 4:**
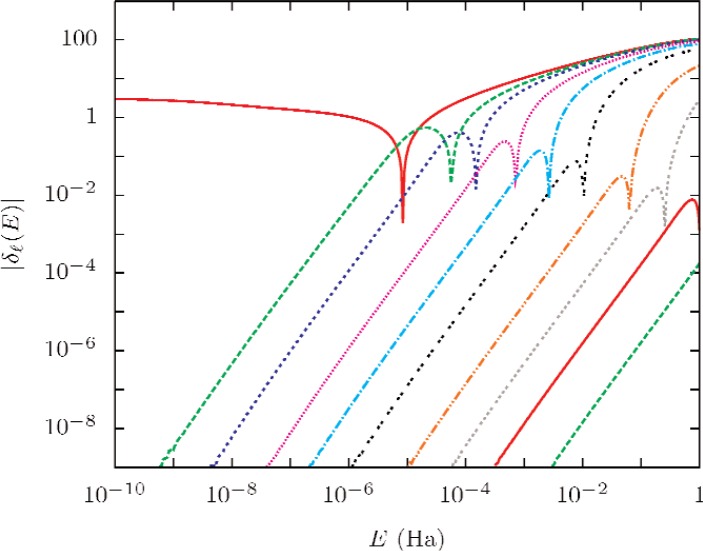
Representative phase shifts as functions of energy. The phase shifts are all positive for small *E*; *δ*_0_ has a zero-*E* limit of π, otherwise *δ_ℓ_*(0) = 0. The lines represent, from left to right, *ℓ* = 0, 2, 4, 10, 20, 40, 100, 200, 400, and 1000.

Figure 4 shows the dependence of the phase shifts on *ℓ* and *E*. It is clear that for small *E*, the sum (9) is dominated by the *ℓ* = 0 term. For larger *E* many terms contribute to the sum. The Born approximation

$$\delta_{B\ell }=-\frac{2\mu \kappa }{m_{e}}\int_{0}^{\infty }\phi (r){\left[j_{\ell }(\kappa r)\right]}^{2}r^{2}dr$$ (12)

(see, e.g. Eq. (38.14) of Ref. [34]) for the phase shift is useful in considering the rate of convergence. For small *κr* the spherical Bessel function can be approximated by the leading term in the Taylor series, (*κr*)*^ℓ^*/(2*ℓ* + 1)!!. The contribution to the integral in Eq. (12) for small *r* thus decreases rapidly with *ℓ*. The spherical Bessel function has a maximum for *κr* near *ℓ* + 1. For larger *ℓ* the Born approximation thus becomes dependent mainly on the weaker attractive part of *ϕ*(*r*) The contributions from power-law potential terms have a simple form:

$$I_{\ell \nu }\equiv \int_{0}^{\infty }{\left[j_{\ell }(\kappa r)\right]}^{2}r^{2-\nu }dr,$$ (13)

which has the values

$$I_{\ell 6}=\frac{3\pi \kappa^{3}}{\left(2\ell -3)(2\ell -1)(2\ell +1)(2\ell +3)(2\ell +5\right)}$$ (14)

and

$$I_{\ell 7}=\frac{4\kappa^{4}}{15(\ell -2)(\ell -1)\ell (\ell +1)(\ell +2)(\ell +3)}.$$ (15)

for the dipole-dipole interaction with and without retardation. The infinite sums of these terms are

$$\sum_{\begin{array}{l} \ell >\ell_{1}\\ \ell \;\text{even} \end{array}}I_{\ell 6}=\frac{3\pi \kappa^{3}}{4\ell_{1}(\ell_{1}+1)(\ell_{1}+2)(\ell_{1}+3)}$$ (16)

$$\sum_{\begin{array}{l} \ell >\ell_{1}\\ \ell \;\text{even} \end{array}}I_{\ell 7}=\frac{\kappa^{4}}{45(2\ell_{1}+1)(2\ell_{1}+3)(2\ell_{1}+5)}.$$ (17)

These can be used to get upper and lower limits for the contributions of the *C*_6_*f*_6_/*r*^6^ term to the truncation error of the sum (9). The following test was made to check the summation error. For *E* ≥ 0.001 hartree, Eq. (12) was used to obtain phase shifts for *ℓ*_1_ = 1000 < *ℓ* ≤ *ℓ*_2_ = 3000, and the corresponding contributions to the sum (9) were evaluated numerically. Equations (16) and (17) were then used with *ℓ*_1_ → *ℓ*_2_ to estimate the truncation error of these numerical sums. The results so obtained were then compared directly with upper and lower limits based on Eqs. (16) and (17). The numerical sums were found to lie very close to the product of *C*_6_ and the right-hand side of Eq. (16). The reason is that asymptotic phase shifts for *ℓ* = 1000 are obtained when *r* is some multiple of 2π/*κ* beyond the first zero of the spherical Bessel function *j*_1000_(*κr*), which occurs near $r=1000.5/\kappa \approx 11.7/\sqrt{E}$. For *E* > 0.001 hartree this is reached before retardation is significant. For smaller *E*, the nodes *r_n_* where Eq. (11) is evaluated occur at larger radii where retardation may be important, but the phase shifts decline sufficiently rapidly with increasing *ℓ* that convergence is obtained for *ℓ* ≪ 1000.

## 4. Virial Coefficients

The second virial coefficient of ^4^He is [33]

$$B=B_{\text{th}}+B_{\text{ideal}}+B_{\text{bound}},$$ (18)

where

$$B_{\text{th}}=-2N_{A}\Lambda^{3}\alpha I_{0}/(\pi T),$$ (19)

$$B_{\text{ideal}}=-N_{A}\Lambda^{3}/16,$$ (20)

$$B_{\text{bound}}=-N_{A}\Lambda^{3}[e^{T_{b}/T}-1],$$ (21)

$$I_{n}=\int_{0}^{\infty }e^{-\alpha \kappa^{2}/T}S(\kappa )\kappa^{n+1}d\kappa ,$$ (22)

and

$$\alpha =(m_{e}/m_{\text{He}})(E_{h}/\kappa_{B}).$$ (23)

In these equations, $\Lambda =\sqrt{2}\lambda_{T}$, where

$$\lambda_{T}=\frac{h}{\sqrt{2m_{\text{He}}k_{B}T}}$$ (24)

is the thermal de Broglie wavelength, and −*T_b_* is the bound state energy in K (Table 4). The temperature derivatives of *B*(*T*) can be evaluated directly from Eqs. (18)–(24). Numerical evaluation of the derivatives requires the integrals *I*_2_ and *I*_4_ in addition to *I*_0_.

The thermal contributions *B*_th_(*T*) require numerical integration over *κ*. The integrals could formally be written with *E* as the integration variable. However, the dependence of the sum terms in the integrand for small *κ* was found to be approximately linear in *κ* ∝ $\sqrt{E}$, so a better spline approximation was obtained by using *κ* as the independent variable.

Formally, the upper limit of integration is infinite. In practice, the phase shifts become increasingly difficult to calculate at higher energies. Calculations were made only up to *E* = 1 hartree. The argument of the exponential factor in the integrand, −*ακ*^2^/T, has a maximum value at *E* = 1 hartree equal to −3.16 × 10^5^ K/*T*, so the integrand is vanishingly small at *κ*_max_, even at T = 10000 K (exp(−31.6) ≈ 1.9 × 10^−14^). The upper limit of integration can thus be safely set at *κ*_max_.

Numerical integrations were required for the integrals *I*_0_, *I*_2_, and *I*_4_. For each case, the sum S(κ) was approximated by cubic splines. The number of knots per decade of energy *E* was 40 for all except *E* > 0.1, where 80 knots were required in order to resolve the rapid dependence of the phase shifts. The integrals were calculated as the sum of a series of integrals with *κ*-limits 0–0.01, 0.01–0.1, 0.1–1, 1–10, and 10−*κ*_max_. This procedure insures sufficient sampling of the integrands, whose peak values depend strongly on *T*.

**Fig. 5:**
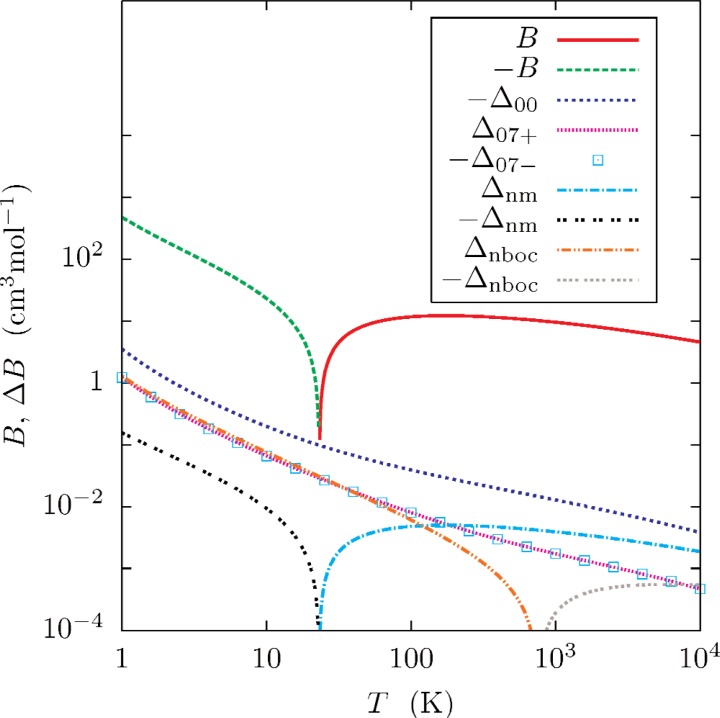
Virial coefficient of ^4^He, calculated under various assumptions. The plots of ±*B*(*T*) = ±*B*_07_(*T*) were calculated with *ϕ*_07_ and atomic masses. The plotted differences are Δ*_x_* = *B_x_* − *B*_07_, where *x* designates the way the virials were calculated; *x* = 00, 07±, and nboc indicates the use of atomic masses and the potentials *ϕ*_00_, *ϕ*_07±_, and *ϕ*_nboc_; *x* = nm indicates calculations with *ϕ*_07_ and nuclear, rather than atomic masses.

**Fig. 6:**
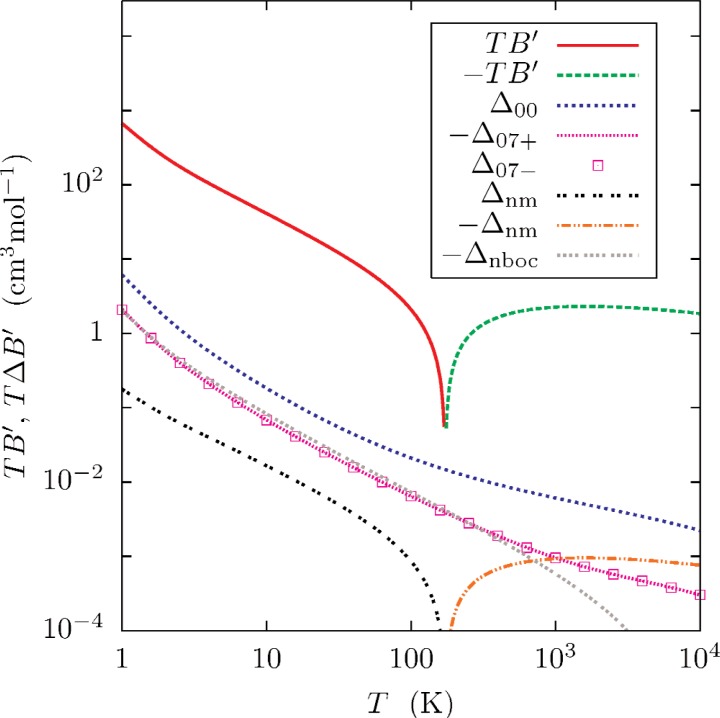
First temperature derivative *B*′(*T*) for ^4^He, plotted as *TB*′. The plotted differences are $\Delta_{x}=T^{2}\left(B_{x}^{\prime }-B_{07}^{\prime }\right)$, where *x* designates the type of calculation (See caption to Fig. 5.)

**Fig. 7:**
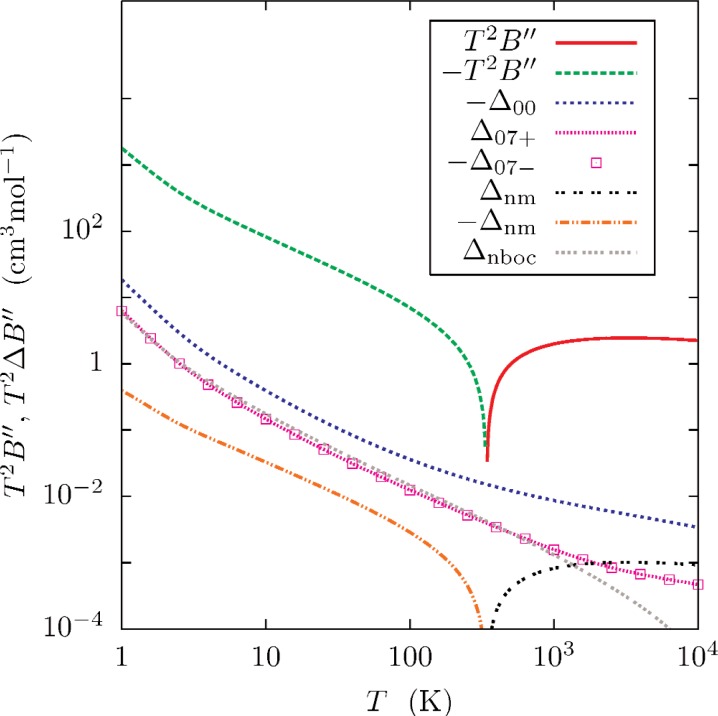
Second temperature derivative *B*″(*T*) for ^4^He, plotted as *T*^2^*B*″. The plotted differences are $\Delta_{x}=T^{2}\left(B_{x}^{\prime \prime }-B_{07}^{\prime \prime }\right)$, where *x* designates the type of calculation (See caption to Fig. 5.)

Figures 5–7 and Table 5 show the virials and the first two temperature derivatives as calculated in this work. Note that the effects of *ϕ*_07±_ on the results is approximately symmetrical. Half of the difference of each calculated property, as computed with *ϕ*_07+_ and *ϕ*_07−_, was chosen as a conservative (*k_u_* ≈ 2) estimate of the uncertainty *U*(*x*) of property *x*. These uncertainty estimates are well-represented by functions of the form

$$k_{u}U(x)=1{\text{cm}}^{3}{\text{mol}}^{-1}\cdot \exp\left[\sum_{n=0}^{4}c_{n}{\left[\ln(T/K)\right]}^{n}\right],$$ (25)

with coefficients listed in Table 6. The table also includes an uncertainty calculation for the acoustic virial

$$\beta_{a}=2B+2(\gamma_{0}-1)TB^{\prime }+{\left(\gamma_{0}-1\right)}^{2}T^{2}B^{\prime \prime }/\gamma_{0},$$ (26)

where *γ*_0_ = 5/3 for helium. Equation (25) represents the uncertainties of *B*, *TB*′, and *T*^2^*B*″ within 2 %, 3 %, and 2 % (rms), respectively, and with a maximum error less than 10 %. The uncertainty of *β_a_* is represented within 2 % (rms) with a maximum error of 5 %. As noted previously, the uncertainty of *ϕ*_07_ has a large coverage factor *k_u_* ≈ 2; a similar coverage factor applies to the uncertainties expressed in Eq. (25) and Table 6.

Figures 5–7 show that that the effects of neglecting the diagonal Born-Oppenheimer correction are no larger than the uncertainties so assigned, and that the effect of using nuclear rather than atomic masses is less than the uncertainties except at the highest temperatures. The differences between values of *B*(*T*) calculated with *ϕ*_07_ and *ϕ*_00_ [1] differ by less than the combined uncertainties (Eq. (25), Table 6 of Ref. [1]).

As noted above, the integrals required for *B*_th_ and its temperature derivatives were done numerically. The automatic integration routine was controlled by specifying an error criterion, which was set sufficiently low that errors in *B* and its derivatives due to the numerical integrations were negligible. This process only insures that the spline-approximated integrand is integrated accurately. It is of more interest to insure that the approximation of the sum term by a spline does not introduce a significant error into the calculation. To estimate this, the number of knots was reduced by eliminating alternate knots, and recalculating *B*(*T*) and its derivatives with the cruder spline approximation. The absolute fractional differences of the two evaluations of *B*(*T*) was less than 3 × 10^−7^ except near the zero of *B*(*T*). The maximum absolute fractional differences of the two evaluations of *TB*′(*T*) was 4 × 10^−7^, and the maximum absolute fractional differences of the two evaluations of *T*^2^*B*″(*T*) was 2 × 10^−6^.

We recommend the use of cubic spline interpolation for estimation of *B*(*T*) between temperatures listed in Table 5. Our tests of such interpolations indicate that the fractional interpolation error is generally much less than 10^−4^ except at the temperature extremes. (Interpolation near the extremes can be improved by using the tabulated higher derivatives to set the end conditions.)

## 5. Viscosity and Thermal Conductivity

The kinetic coefficients depend on the quantum cross-sections [30] defined by

$$Q^{\left(2\right)}=\frac{8\pi }{\kappa^{2}}\sum_{\ell =0}^{\infty }\frac{\left(2\ell +1\right)\left(\ell +2\right)}{2\ell +3}\sin^{2}\left(\delta_{\ell }-\delta_{\ell +2}\right),$$ (27)

$$\begin{array}{l} Q^{\left(4\right)}=\frac{8\pi }{{}^{\kappa^{2}}}\sum_{\ell =0}^{\infty }[\frac{2\left(\ell +1\right)\left(\ell +2\right)\left(2\ell^{2}+6\ell -3\right)}{\left(2\ell -1\right)\left(2\ell +3\right)\left(2\ell +7\right)}\sin^{2}\left(\delta_{\ell }-\delta_{\ell +2}\right)\\ \;+\frac{\left(\ell +1\right)\left(\ell +2\right)\left(\ell +3\right)\left(\ell +4\right)}{\left(2\ell +3\right)\left(2\ell +5\right)\left(2\ell +7\right)}\sin^{2}\left(\delta_{\ell }-\delta_{\ell +4}\right)], \end{array}$$ (28)

and

$$\begin{array}{l} Q^{\left(6\right)}=\frac{8\pi }{\kappa^{2}}\sum_{\ell =0}^{\infty }[\frac{15\left(\ell +1\right)\left(\ell +2\right)\left(\ell^{4}+6\ell^{3}+\ell^{2}-24\ell +9\right)}{\left(2\ell -3\right)\left(2\ell -1\right)\left(2\ell +3\right)\left(2\ell +7\right)\left(2\ell +9\right)}\sin^{2}\left(\delta_{\ell }-\delta_{\ell +2}\right)\\ \;+\frac{3\left(\ell +1\right)\left(\ell +2\right)\left(\ell +3\right)\left(\ell +4\right)\left(2\ell^{2}+10\ell -5\right)}{\left(2\ell -1\right)\left(2\ell +3\right)\left(2\ell +5\right)\left(2\ell +7\right)\left(2\ell +11\right)}\sin^{2}\left(\delta_{\ell }-\delta_{\ell +4}\right)\\ \;+\frac{\left(\ell +1\right)\left(\ell +2\right)\left(\ell +3\right)\left(\ell +4\right)\left(\ell +5\right)\left(\ell +6\right)}{\left(2\ell +3\right)\left(2\ell +5\right)\left(2\ell +7\right)\left(2\ell +9\right)\left(2\ell +11\right)}\sin^{2}\left(\delta_{\ell }-\delta_{\ell +6}\right)]. \end{array}$$ (29)

Equations (27)–(29) converge rapidly; numerical evaluation was straightforward. The collision integrals needed for computation of kinetic coefficients are expressed in terms of normalized cross sections, defined for even *n* > 0 by

$$Q^{\left(n\right)*}\equiv \frac{Q^{\left(n\right)}}{\pi r_{m}^{2}n/\left(n+1\right)},$$ (30)

where *r_m_* (actually an arbitrary length) is the radial position of the potential minimum. The collision integrals are defined as

$$\Omega^{\left(n,s\right)*}\equiv \frac{\int_{0}^{\infty }Q^{\left(n\right)*}\left(E\right)e^{-\beta E}E^{s+1}dE}{\left(s+1\right)!{\left(k_{B}T\right)}^{s+2}},$$ (31)

where *β* ≡ *E_h_*/(*k_B_T*). Collision integrals with *n* = 2, *s* = 2, 4, … 10; *n* = 4; *s* = 4, 6, 8; and *n* = 6, *s* = 6 are needed for the fifth-order calculation of viscosity and thermal conductivity [35]. The collision integrals were calculated by using cubic spline representations of the collision integrals, and dividing the integrals in Eq. (31) into 11 sub-intervals, with limits 0–10^−10^, 10^−10^–10^−9^, … 0.1–1. This division insured adequate sampling of the integrands, whose peak locations vary rapidly with temperature. (The errors introduced by truncating the infinite integral are neglibible.)

The viscosity is [35,36]

$$\eta =\frac{5\sqrt{\pi mk_{B}T}}{16\pi r_{m}^{2}\Omega^{\left(2,2\right)*}}f_{\eta }^{\left(n\right)},$$ (32)

where $f_{\eta }^{\left(n\right)}$ is obtained by solving a set of linear equations

$$B\xi =\left(\begin{array}{ccccc} b_{11} & b_{12} & b_{13} & b_{14} & b_{15}\\ b_{21} & b_{22} & b_{23} & b_{24} & b_{25}\\ b_{13} & b_{32} & b_{33} & b_{34} & b_{35}\\ b_{14} & b_{24} & b_{34} & b_{44} & b_{45}\\ b_{15} & b_{25} & b_{35} & b_{45} & b_{55} \end{array}\right)\left(\begin{array}{c} \xi_{1}\\ \xi_{2}\\ \xi_{3}\\ \xi_{4}\\ \xi_{5} \end{array}\right)=\left(\begin{array}{c} 1\\ 0\\ 0\\ 0\\ 0 \end{array}\right)=e_{1}$$ (33)

for $\xi_{1}\equiv f_{\eta }^{\left(n\right)}/b_{11}$. The components of the symmetric matrix **B** are listed in Appendix A of Ref. [35]. In particular, since *b*_11_ = 4Ω^(2,2)*^, the viscosity can be expressed as

$$\eta =\frac{5\sqrt{\pi mk_{B}T}}{4\pi r_{m}^{2}}\xi_{1}.$$ (34)

Similarly, the thermal conductivity can be determined from the solution of

$$A\zeta =e_{1},$$ (35)

where the components of **A** are defined in Appendix B of Ref. [35], and ***ζ*** is a column vector with components *ζ_j_*. The thermal conductivity depends only on *ζ*_1_:

$$\lambda =\frac{75k_{B}\sqrt{\pi mk_{B}T}}{16mr_{m}^{2}}\zeta_{1}.$$ (36)

To insure that the complicated formulas for the components of B (and the corresponding matrix for the thermal conductivity) were transcribed accurately, the following procedure was followed. The formulas were extracted from an electronic copy of Ref. [35]. These were further edited to conform with Fortran notation. Subsequently, Viehland provided Fortran codes that generated the Fortran code for calculating the matrices directly [37]. Numerical evaluations using the two implementations were identical within machine precision.

Errors in the numerical integrations required for calculating *η* and *λ* were estimated by eliminating alternate knots in the spline representations of the collision integrals and repeating the calculations. The two values of *η*(*T*), and the two values of *λ*(*T*) so determined had an absolute fractional difference of less than 3 × 10^−6^ at *T* = 1 K. This difference declined with *T* and remained below 1.2 × 10^−7^ for *T* > 20 K.

**Fig. 8:**
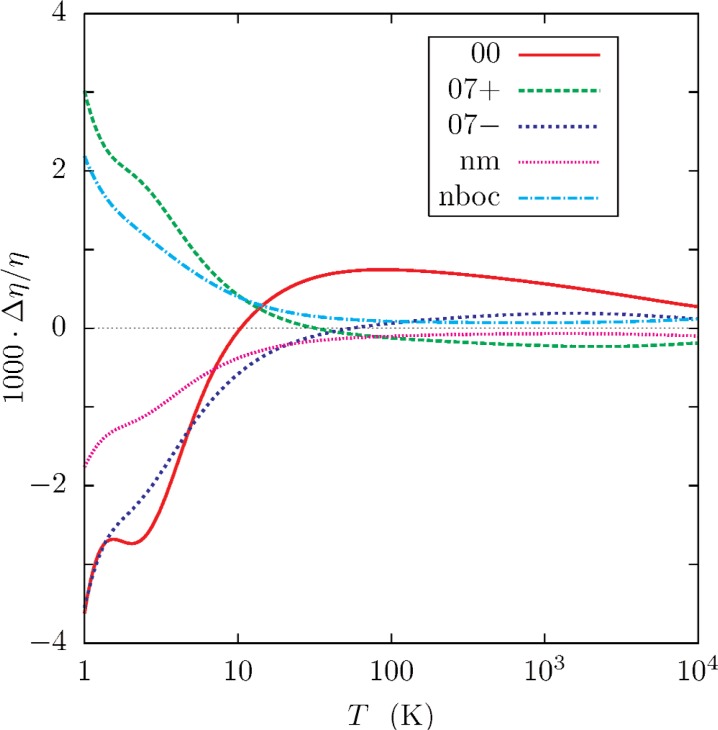
Sensitivity of the viscosity of ^4^He to various options in the calculations. The fractional difference between *η_x_* and the value calculated with *ϕ*_07_ and atomic masses is plotted as the fraction Δ*η*/*η* = (*η_x_* − *η*_07_)/*η*_07_, where *x* specifies the type of calculation (See caption to Fig. 5.) A similar plot for the thermal conductivity differs from this plot only in minor details.

The viscosities and thermal conductivities determined in this work are listed in Table 5. Figure 8 and a nearly identical figure for Δ*λ*/*λ* show the sensitivity of the calculations to the choice of potential and the choice of nuclear instead of atomic masses. The effects of using potential *ϕ*_07±_ is nearly symmetric. Half of the differences between values of *η* or *λ* calculated with these two potentials approaches 0.35 % at low temperature. The differences reverse sign near 42 K. Above this temperature, the half-difference is bound by 0.02 %. A reasonable estimate of the the relative uncertainty *U_r_* in either *η* or *λ* is the minimum of 0.35 % and the equation

$$k_{u}U_{r}(\eta )=k_{u}U_{r}(\lambda )=0.0002+0.005K/T.$$ (37)

Values of the viscosity and thermal conductivity at temperatures between those listed in Table 5 can be obtained by interpolating with cubic splines. Our tests indicate that cubic spline interpolation introduces a fractional error of less than 10^−5^ except near the temperature extremes.

## 6. Validation of Computations

The Fortran code used for calculating the phase shifts and for subsequent calculation of the thermo-physical properties was tested by an independent development of new codes by one of us (Mehl) to test the results of Hurly and Moldover [1]. The test demonstrated excellent agreement of the sum (9) and the quantum cross-sections (27)–(29).

The test revealed two errors in the calculation of the thermophysical properties reported in Ref. [1]. The first was an incorrect sign assigned to the bound-state contribution to the published virials, which mainly affected the low temperature results for ^4^He and for ^3^He-^4^He mixtures. The second was due to inconsistent units conversion. The code used by Hurly to calculate the thermal conductivity was based on the equivalent of Eq. (36) in Hirschfelder *et al.* [36]. Their Eq. (8.2–31) uses a calorie unit in a numerical prefactor. Conversion of this to J using a current definition of the calorie introduced a factor of 1.000545 error in the thermal conductivity results published in Ref. [1]. The published values are high by this factor.

## 7. Comparisons With Recent Experiments

**Fig. 9:**
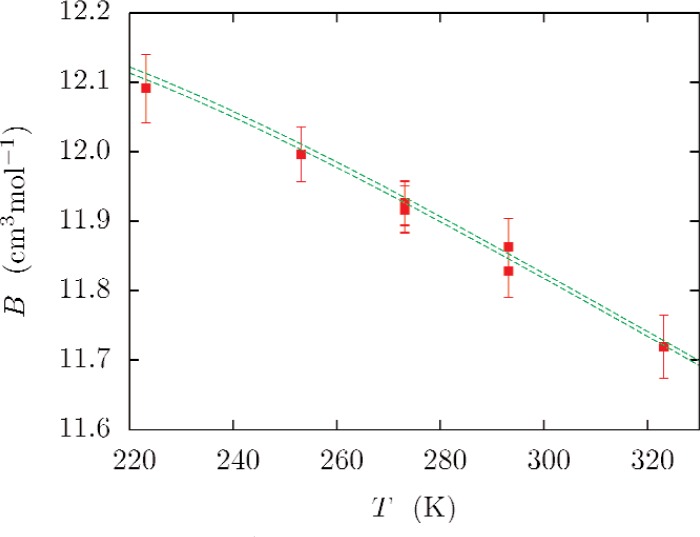
Second virial coefficients of ^4^He measured by McLinden *et al.* [38], compared with values calculated with *ϕ*_07±_ (dashed lines). Values calculated with *ϕ*_07_ fall between the dashed lines. The error bars indicate the experimental *k_u_* = 1 uncertainties.

Hurly and Moldover [1] compared their results with a wide range of experimental results. Here we limit our comparisons to a few accurate experiments published since 2000. Figure 9 compares the recent second virial measurements of McLinden and Lösch-Will [38]. The agreement is excellent.

**Fig. 10:**
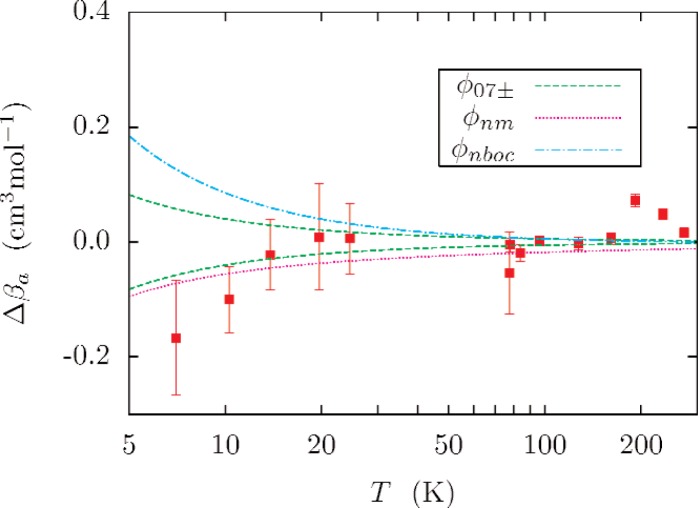
Acoustic virial coefficient of Pitre *et al.* [5] compared with values calculated with *ϕ*_07_; Δ*β_a_* = *β_a_*,_expt_ − *β_a_*_,calc_. The dashed lines are plots of *β_a_*_,07±_ − *β_a_*_,07_, and indicate the uncertainty of the theoretical calculation. The error bars indicate the experimental (*k_u_* = 1) uncertainties. Other lines show Δ*β_a_* corresponding to *ϕ*_nm_, and *ϕ*_nboc_. The acoustic virial is clearly sensitive to the differences between the various potentials.

Figure 10 compares the recent measurements of the acoustic virial by Pitre, Moldover and Tew [5]. The measurements fall well within the combined (*k_u_* = 2) uncertainty of the predicted slope *β_a_* and the experimental uncertainty except at high temperatures, where the disagreement is on the order of the scatter in the measurements.

Berg’s high quality measurement of the viscosity [39,40] at 298.15 K (expressed with a *k_u_* = 2 uncertainty), (19.842 ± 0.014) *µ*Pa·s, and the calculated value (19.824 ± 0.004) *µ*Pa·s differ by the sum of their *k_u_* = 2 uncertainties.

## 8. Concluding Remarks

As shown in Fig. 3, multiple research groups have provided us with very accurate ab initio “data” at 4.0 and 5.6 bohr. In order to fully exploit these data, it would be desirable to have theoretical potentials of similar accuracy at nearby *r*. The most demanding gas metrology is conducted near 273 K; thus, it would be very desirable to generate ab initio values of the potential at, for example *r* = 3.89 and 4.13 bohr (corresponding to *ϕ* = 200 K and 450 K) with uncertainties comparable to those already achieved at 4.0 and 5.6 bohr.

## Figures and Tables

**Table 1:** Attractive interaction coefficients [11] for helium atoms with ^4^He and infinite mass nucleii

	^4^He	^∞^He
*C*_6_ (hartree-bohr^6^)	1.462122853192	1.460977837725
*C*_8_ (hartree-bohr^8^)	14.12578806	14.11785737
*C*_10_ (hartree-bohr^10^)	183.781468	183.691075
*C*_12_ (hartree-bohr^12^)	3267.13274	3265.256092
*C*_14_ (hartree-bohr^14^)	76501.2887	76571.26764
*C*_16_ (hartree-bohr^16^)	2277412.86	2276292.717

**Table 2:** Variable (fit) potential coefficients

Potential	*a*_−2_ (bohr^2^)	*a*_−1_ (bohr)	*a*_0_ (−)	*a*_1_ (bohr^−1^)	*a*_2_ (bohr^−2^)	*δ* (bohr^−1^)
*ϕ*_07_	0.081212	−0.28755	2.14735	−1.97272	−0.051787	1.992657
*ϕ*_07−_	0.097486	−0.32441	2.17654	−1.98206	−0.050505	2.006175
*ϕ*_07+_	0.065002	−0.25089	2.11837	−1.96343	−0.053050	1.980020
*ϕ*_nboc_	0.072490	−0.26814	2.13133	−1.96754	−0.052524	1.985551

**Table 3:** Theoretical potential values used to determine *ϕ*_07_. The model potential *ϕ*_07_ was fit to the sum of the theoretical potential *ϕ* and the diagonal Born-Oppenheimer correction Δ*ϕ*_DBOC_ with weighting equal to the inverse square of the uncertainty *U*(*ϕ*). When a single source is listed, the uncertainty is generally that stated in the source. When multiple sources are cited, the unweighted mean and standard deviation of the set is used. In some cases, indicated by an asterisk, the uncertainty was adjusted upward to account for disagreement with neighboring values.

*r* (bohr)	*ϕ* (K)	*U*(*ϕ*) (K)	Δ*ϕ*_DBOC_ (K)	Source(s)
1	286435	25	158	[19]
1.5	104320	20	36	[19]
2	36144.6	10	11.8	[19]
2.5	11962.0	1.0	4.1	[19,23]^*^
3	3786.0	20	1.37	[17,19,23,24]
3.5	1111.0	1.0	0.41	[19,20,23]^*^
4	292.64	0.10	0.10	[20–23,25,26]
4.5	58.400	0.10	0.009	[19,20,23,24]
5	−0.500	0.10	−0.013	[19,20,23,24]
5.1	−4.534	0.025	−0.014	[20,23]
5.6	−10.991	0.011	−0.012	[20–26]
6	−9.671	0.009	−0.011	[20,23]^*^
6.5	−6.887	0.005	−0.008	[23]
6.6	−6.340	0.020	−0.007	[20,24]
7	−4.619	0.007	−0.005	[26]
7.5	−3.073	0.005	−0.004	[20,23,25]^*^
8	−2.066	0.002	−0.002	[23]^*^
9	−0.989	0.001	−0.002	[25]
10	−0.5130	0.0002	−0.001	[23]^*^
12	−0.166	0.0010	0.000	[25]
15	−0.0423	0.0002	0.000	[25]

**Table 4:** The potential minima *ϕ*_min_ = *ϕ*(*r_m_*) for the potentials used in this work, and the corresponding bound-state energies. (The retardation corrections *f*_2_*_n_* were included in these calculations.)

Potential	*ϕ*_min_ (K)	*r_m_* (bohr)	He mass	*E*_bound_ (mK)
*ϕ*_07_	−10.999	5.608	Atomic	−1.555
*ϕ*_07−_	−10.983	5.608	Atomic	−1.667
*ϕ*_07+_	−11.014	5.607	Atomic	−1.438
*ϕ*_nboc_	−10.985	5.608	Atomic	−1.550
*ϕ*_07_	−10.999	5.608	Nuclear	−1.520

**Table 5:** Thermophysical properties of ^4^He calculated in this work. Calculated quantities are printed with at least one excess figure as an aid in smooth interpolation; for the uncertainties of *B* and its derivatives use Eqs. (25) and Table 6; for the uncertainties of *η* and *λ* use Eq. (37).

*T* (K)	*B* (cm^3^mol^−1^)	*TB*′ (cm^3^mol^−1^)	*T*^2^*B*″ (cm^3^mol^−1^)	*η* (*µ*Pa·s)	*λ* (mWm^−1^K^−1^)
1.0	−475.05	669.19	−1790.42	0.3292	2.632
1.2	−369.75	495.66	−1294.92	0.3405	2.720
1.4	−301.99	388.71	−986.65	0.3583	2.845
1.6	−254.99	318.11	−783.24	0.3844	3.033
1.8	−220.53	268.92	−642.77	0.4183	3.283
2.0	−194.14	233.07	−542.07	0.4586	3.588
2.25	−168.70	200.19	−451.94	0.5161	4.030
2.5	−148.92	175.86	−387.37	0.5791	4.519
2.75	−133.07	157.15	−339.36	0.6457	5.037
3.0	−120.06	142.28	−302.48	0.7141	5.569
3.5	−99.90	120.06	−249.71	0.8511	6.637
4.0	−84.96	104.14	−213.73	0.9834	7.666
4.5	−73.42	92.10	−187.46	1.1078	8.636
5.0	−64.23	82.63	−167.31	1.2239	9.542
6.0	−50.48	68.63	−138.19	1.4339	11.184
7.0	−40.683	58.72	−117.98	1.6209	12.650
8.0	−33.346	51.328	−103.05	1.7919	13.992
9.0	−27.646	45.582	−91.55	1.9514	15.244
10	−23.090	40.984	−82.39	2.1023	16.427
11	−19.366	37.216	−74.93	2.2463	17.556
12	−16.267	34.069	−68.73	2.3846	18.641
14	−11.407	29.105	−58.988	2.6472	20.699
16	−7.776	25.358	−51.679	2.8947	22.638
18	−4.965	22.423	−45.981	3.1299	24.481
20	−2.729	20.060	−41.408	3.3550	26.244
22	−0.911	18.113	−37.653	3.5715	27.939
23	−0.125	17.262	−36.014	3.6768	28.764
24	0.592	16.479	−34.509	3.7804	29.575
25	1.250	15.757	−33.121	3.8824	30.374
26	1.855	15.088	−31.837	3.9828	31.160
28	2.928	13.888	−29.537	4.1795	32.700
30	3.850	12.841	−27.534	4.3709	34.199
35	5.663	10.729	−23.496	4.8301	37.793
40	6.986	9.123	−20.432	5.2659	41.204
45	7.985	7.860	−18.024	5.6828	44.466
50	8.758	6.838	−16.077	6.0837	47.603
60	9.860	5.286	−13.117	6.8465	53.570
70	10.586	4.1591	−10.967	7.5673	59.208
80	11.0827	3.3033	−9.329	8.2549	64.585
90	11.4314	2.6306	−8.038	8.9149	69.746
100	11.6795	2.0876	−6.994	9.5519	74.726
120	11.9830	1.2650	−5.403	10.7689	84.240
140	12.1311	0.6715	−4.2464	11.9245	93.272
160	12.1903	0.2235	−3.3667	13.0310	101.919
180	12.1956	−0.1262	−2.6743	14.0968	110.248
200	12.1673	−0.4063	−2.1150	15.1284	118.308
225	12.1026	−0.6863	−1.5506	16.3769	128.062
250	12.0183	−0.9096	−1.0953	17.5862	137.510
273.16	11.9301	−1.0791	−0.7458	18.6765	146.027
275	11.9228	−1.0913	−0.7205	18.7620	146.695
298.15	11.8289	−1.2315	−0.4280	19.8245	154.994
300	11.8212	−1.2418	−0.4065	19.9084	155.649
325	11.7167	−1.3680	−0.1398	21.0288	164.400
350	11.6113	−1.4752	0.0893	22.1260	172.970
375	11.5063	−1.5671	0.2883	23.2024	181.375
400	11.4026	−1.6465	0.4626	24.2598	189.633
450	11.2008	−1.7763	0.7531	26.3241	205.753
500	11.0082	−1.8771	0.9850	28.3298	221.413
600	10.6523	−2.0209	1.3306	32.1959	251.596
700	10.3332	−2.1157	1.5740	35.9049	280.550
800	10.0462	−2.1802	1.7529	39.4880	308.517
900	9.7867	−2.2251	1.8887	42.9671	335.670
1000	9.5505	−2.25660	1.9943	46.3584	362.135
1200	9.1354	−2.29376	2.1455	52.924	413.367
1400	8.7804	−2.31003	2.2454	59.256	462.771
1600	8.4715	−2.31430	2.3139	65.402	510.71
1800	8.1990	−2.31131	2.3617	71.395	557.46
2000	7.9559	−2.30378	2.39552	77.259	603.20
2500	7.4448	−2.27457	2.44221	91.470	714.02
3000	7.03314	−2.23916	2.45852	105.185	820.95
3500	6.69073	−2.20239	2.45921	118.527	924.97
4000	6.39902	−2.16616	2.45129	131.578	1026.70
4500	6.14591	−2.13125	2.43847	144.394	1126.60
5000	5.92310	−2.09794	2.42281	157.018	1224.99
6000	5.54615	−2.03623	2.38740	181.810	1418.18
7000	5.23652	−1.98063	2.35026	206.135	1607.72
8000	4.97538	−1.93035	2.31346	230.116	1794.54
9000	4.75070	−1.88461	2.27786	253.835	1979.31
10000	4.55433	−1.84276	2.24378	277.355	2162.52

**Table 6:** Coefficients in Eq. (25) for estimating the uncertainty of *B*(*T*) and its temperature derivatives.

Property	*c*_0_	*c*_1_	*c*_2_	*c*_3_	*c*_4_
*B*	0.1341	−1.4474	0.0960	−0.00327	–
*TB*′	0.6612	−1.8415	0.2173	−0.02476	0.00128
*T*^2^*B*″	1.8238	−2.2109	0.3379	−0.04263	0.002166
*β_a_*	0.2661	−1.4560	0.1134	−0.00479	–

## References

[1] Hurly JJ, Moldover MR (2000). Ab initio values of the thermo-physical properties of helium as standards. J Res Natl Inst Stand Technol.

[2] Handy Nicholas C, Lee Aaron M (1996). The adiabatic approximation. Chem Phys Lett.

[3] Kutzelnigg Werner (1997). The adiabatic approximation: I. The physical background of the Born-Handy ansatz. Mol Phys.

[4] Rusby RL, Hudson RP, Durieux M, Schooley JF, Steur PPM, Swenson CA (1991). Thermodynamic basis of the ITS-90. Metrologia.

[5] Pitre Laurent, Moldover Michael R, Tew Weston L (2006). Acoustic thermometry: new results from 273 K to 77 K and progress towards 4 K. Metrologia.

[6] May Eric F, Moldover Michael R, Berg Robert F, Hurly John J (2006). Transport properties of argon at zero density from viscosity-ratio measurements. Metrologia.

[7] Fellmuth Bernd, Gaiser Christof, Fischer Joachim (2006). Determination of the Boltzmann constant – status and prospects. Meas Sci Technol.

[8] Gavioso R, May Eric F, Schmidt James W, Moldover Michael R, Wang Y (2006). Towards an electrical pressure standard: Dielectric permittivity of helium and argon measured with quasi-spherical microwave resonators and cross capacitors.

[9] Mohr Peter J, Taylor Barry N (2005). CODATA recommended values of the fundamental physical constants: 2002. Rev Mod Phys.

[10] Tang KT, Toennies JP (1984). An improved simple model for the van der Waals potential based on universal damping functions for the dispersion coefficients. J Chem Phys.

[11] Zhang J-Y, Yan Z-C, Vrinceanu D, Babb JF, Sadeghpour HR (2006). Long-range interactions for He(*ns*)-He(*n*′*s*) and He(*ns*)-He(*n*′*p*). Phys Rev A.

[12] Thakkar Ajit J (1988). Higher dispersion coefficients: Accurate values for hydrogen atoms and simple estimates for other systems. J Chem Phys.

[13] Bishop David M, Pipin Janusz (1993). Dipole, quadrupole, octupole, and dipole-octupole polarizabilities at real and imaginary frequencies for H, He, and H_2_ and the dispersion-energy coefficients for interactions between them. Int J Quant Chem.

[14] Chen Ming-Keh, Chung Kwong T (1996). Retardation long-range potentials between two helium atoms. Phys Rev A.

[15] Jamieson MJ, Drake GWF, Dalgarno A (1995). Retarded dipole-dipole dispersion interaction potential for helium. Phys Rev A.

[16] Komasa Jacek, Cencek Wojciech, Rychlewski Jacek (1999). Adiabatic corrections of the helium dimer from exponentially correlated gaussian functions. Chem Phys Lett.

[17] Ceperley DM, Partridge H (1986). The He2 potential at small distances. J Chem Phys.

[18] Korona T, Williams HL, Bukowski R, Jeziorski B, Szalewicz K (1997). Helium dimer potential from symmetry-adapted perturbation theory calculations using large gaussian geminal and orbital basis sets. J Chem Phys.

[19] Komasa Jacek (1999). Exponentially correlated gaussian functions in variational calculations: Energy expectation values in the ground state helium dimer. J Chem Phys.

[20] van Mourik Tanja, Dunning Thom H (1999). A new ab initio potential energy curve for the helium dimer. J Chem Phys.

[21] van de Bovenkamp J, van Duijneveldt FB (1999). MRCI calculations on the helium dimer employing an interaction optimized basis set. J Chem Phys.

[22] Klopper Wim (2001). A critical note on extrapolated helium pair potentials. J Chem Phys.

[23] Gdanitz Robert J (2001). Accurately solving the electronic Schrödinger equation of atoms and molecules using explicitly correlated (*r*_12−_)MR-CI. VI. The helium dimer (He_2_) revisited. Mol Phys.

[24] Anderson James B (2001). An exact quantum monte carlo calculation of the helium-helium intermolecular potential II. J Chem Phys.

[25] Anderson James B (2004). Comment on An exact quantum monte carlo calculation of the helium-helium intermolecular potential. J Chem Phys.

[26] Cencek Wojciech, Jeziorska Malgorzata, Bukowski Robert, Jaszuński Michal, Jeziorski Bogumil, Szalewicz Krzysztof (2004). Helium dimer interaction energies from Gaussian geminal and orbital calculations. J Phys Chem A.

[27] Cencek Wojciech, Komasa Jacek, Pachucki Krzysztof, Szalewicz Krzysztof (2005). Relativistic correction to the helium dimer interaction energy. Phys Rev Lett.

[28] Pachucki Krzysztof, Komasa Jacek (2004). Radiative corrections to the helium dimer interaction energy. Phys Rev Lett.

[29] Taylor Barry N, Kuyatt Chris E, NIST Technical Note 1297 (1994). Guidelines for Evaluating and Expressing the Uncertainty of NIST Measurements.

[30] Meeks FR, Cleland TJ, Hutchinson KE, Taylor WL (1994). On the quantum cross sections in dilute gases. J Chem Phys.

[31] Munn RJ, Mason EA, Smith FJ (1964). Some aspects of the quantal and semi-classical calculation of phase shifts and cross sections for molecular scattering and transport. J Chem Phys.

[32] Hepburn JW, Le Roy RJ (1978). On calculating phase shifts and performing fits to scattering cross sections or transport properties. Chem Phys Lett.

[33] Kirkpatrick John E, Keller William E, Hammel Edward F, Metropolis Nicholas (1954). Second virial coefficients of He^3^ and He^4^. Phys Rev.

[34] Schiff Leonard I (1968). Quantum Mechanics.

[35] Viehland Larry A, Janzen Alec R, Aziz Ronald A (1995). High approximations to the transport properties of pure atomic gases. J Chem Phys.

[36] Hirschfelder Joseph O, Bird R Byron, Curtiss Charles F (1954). Molecular Theory of Gases and Liquids.

[37] Viehland Larry A Private communication of the codes vis.alg and th.con.alg referred to in Ref. [35].

[38] McLinden Mark O, Lösch-Will Cornelia (2007). Apparatus for wide-ranging, high-accuracy fluid (*p, ρ, T*) measurements based on a compact two-sinker densimeter. J Chem Thermodyn.

[39] Berg Robert F (2005). Simple flow meter and viscometer of high accuracy for gases. Metrologia.

[40] Berg Robert F (2006). Erratum. Metrologia.

