# Errata

**DOI:** 10.6028/jres.112.014

**Published:** 2007-06-01

***Erratum:*** *See below: correct description of Cover art for Volume 112, Number 2, March–April 2007.*

Symbol in line 2 should be as follows: potential *ϕ*_07_, ..…

Symbol in line 5 should be as follows: uncertainty of *ϕ*_07_ and..…

[J. Res. Natl. Inst. Stand. Technol. Volume 112, Number 2, March-April 2007]

**Cover:** The cover art depicts a Helium atom which is the subject of improved ab initio calculations for He-He interactions. These improvements have led to (1) the construction of a new interatomic potential *ϕ*_07_, (2) recalculation of values for the second virial coefficient, the viscosity, and the thermal conductivity of ^4^He from 1 K to 10,000 K, and (3), the analysis of the uncertainties of the thermophysical properties that propagate from the uncertainty of *ϕ*_07_ and from the Born-Oppenheimer approximation of the electron-nucleon quantum mechanical system. See the paper by Hurly and Mehl in this issue (pages 75–84) for the complete details of these calculations. Cover art by C. Carey.

